# Inhibition of urethral stricture by a catheter loaded with nanoparticle/ pirfenidone complexes

**DOI:** 10.3389/fbioe.2023.1254621

**Published:** 2023-10-26

**Authors:** Wei Meng, Zhaosheng Jiang, Jiahao Wang, Xiaohua Chen, Bo Chen, Bo Cai, Youlang Zhou, Limin Ma, Yangbo Guan

**Affiliations:** ^1^ Department of Urology, Affiliated Hospital of Nantong University, Medical School of Nantong University, Nantong, China; ^2^ Department of Urology, Nantong TCM Hospital Affiliated to Nanjing University of Chinese Medicine, Nantong, China; ^3^ Department of Urology, Wuxi Hospital Affiliated to the Nanjing University of Chinese Medicine, Wuxi, China; ^4^ Department of Imaging, Affiliated Hospital of Nantong University, Medical School of Nantong University, Nantong, China; ^5^ Research Central of Clinical Medicine, Affiliated Hospital of Nantong University, Medical School of Nantong University, Nantong, China

**Keywords:** pirfenidone, nanoparticle, urethral stricture, catheter, fibrosis

## Abstract

**Background:** Urethral strictures are common injurious conditions of the urinary system. Reducing and preventing urethral strictures has become a hot and challenging topic for urological surgeons and related researchers. In this study, we developed a catheter loaded with nanoparticle/pirfenidone (NP/PFD) complexes and evaluated its effectiveness at inhibiting urethral stricture in rabbits, providing more references for the clinical prevention and reduction of urethral stenosis.

**Methods:** Twelve adult male New Zealand rabbits were selected and divided into the following four groups in a ratio of 1:1:1:1 using the random number table method: Group A, sham; Group B, urethral stricture (US); Group C, US + unmodified catheter; and Group D, US + NP/PFD catheter. On the 30th day after modelling, retrograde urethrography was performed to evaluate urethral stricture formation, and histopathological examination was performed on the tissues of the corresponding surgical site. Meanwhile, changes in the expression level of Transforming growth factor β1 (TGF-β1) in the tissues were detected by immunohistochemistry.

**Results:** The NP/PFD complexes adhered uniformly to the catheter surface. They remained on the surface of the catheter after insertion into the urethra. In addition, the NP/PFD complexes spread into the urethral epithelium 2 weeks after surgery. Ultimately, urethral strictures were significantly reduced with the placement of the NP/PFD complex catheter.

**Conclusion:** Our catheter loaded with NP/PFD complexes effectively delivered PFD to the urethral epithelium through continuous local delivery, thereby reducing fibrosis and stricture after urethral injury, which may be associated with the inhibition of TGF-β1 expression.

## Introduction

Narrowing of the urethral lumen due to ischaemic spongiofibrosis promotes urethral stricture formation. The incidence of urethral strictures is approximately 0.6% in males (Mann et al., 2021). The main causes of urethral strictures include external urethral injury and internal urethral injury, such as pelvic fracture., with the latter mostly due to iatrogenic injury, such as mechanical injury, thermal injury, and laser energy radiation injury caused by endourological procedures (Verla et al., 2019; Mangir and Chapple, 2020). With gradual narrowing of the urethral lumen, these patients may present with different degrees of lower urinary tract obstruction symptoms, such as urinary hesitancy, incomplete emptying, nocturnal urination, and frequent urination (Hong et al., 2019), and in severe cases, urinary retention, urethral infection/necrosis, periurethral abscess, and renal failure.

The currently avaialble treatment mainly includes urethral dilation, urethral incision, urethral stent placement, and urethral reconstruction surgery (such as stenosis resection, urethral end-to-end anastomosis, oral mucosa replacement urethroplasty, and tissue engineering replacement urethroplasty) (King and Rourke, 2019; Tritschler and Beck, 2021). However, despite the rapid development of urethroplasty, the recurrence rate remains high because of submucosal fibrosis and scarring of the urethra after replacement surgery (Chhetri et al., 2009; Barbagli and Lazzeri, 2011), particularly in patients with long-distance urethral strictures, who often require regular urethral dilation or even re-surgery (Astolfi et al., 2019; Campos-Juanatey et al., 2020). This places great physical, psychological, and economic burdens on the patients. To this end, many studies have addressed the principles of cell-based therapy, drug delivery, and tissue engineering, providing more potential options for the treatment of urethral stricture (Farzamfar et al., 2022).

The formation and recurrence of urethral strictures are related to the formation of fibrosis in the healing process of the injured tissue, which is mainly related to the excessive deposition of extracellular matrix (ECM) in the submucosa of the urethra, an increase in collagen level, a change in the proportion of collagen types I and III, and an imbalance in ECM degradation (Hughes et al., 2020; Feng et al., 2021). Similar to many fibrosis-related diseases, abnormal expression of fibroblast genes in urethral tissues is believed to be a major cause of scarring (Ding et al., 2022). Transforming growth factor β1 (TGF-β1) is an effective chemokine of fibroblasts and an important mediator of fibrosis, as it induces the production and secretion of collagen by fibroblasts, thereby causing tissue fibrosis (Zhang et al., 2022). Therefore, the effective application of antifibrosis drugs has become an important strategy to treat urethral strictures.

Pirfenidone (PFD) has been clinically used for the treatment of idiopathic pulmonary fibrosis. As a TGF-β ligand inhibitor, PFD inhibits fibroblast proliferation and collagen synthesis by blocking the production of TGF-β in a variety of cells, thus slowing the progression of idiopathic fibrosis. The antifibrotic effects of PFDs have been demonstrated in basic and clinical studies in a variety of disease models, including acute kidney injury, Crohn’s disease, and COVID-19 (Latella and Viscido, 2020; Pirfenidone, 2020; Ruwanpura et al., 2020; Sharawy and Serrya, 2020). Therefore, we selected PFD as an antifibrosis drug to explore its effectiveness in the treatment of urethral strictures.

Over the past 10 years, materials and tissue engineering fields have developed rapidly. Numerous studies have shown that nanomaterials can be used for tissue engineering, reconstruction, and the controlled release of drugs (Bhattarai et al., 2018; Hemamalini and Giri Dev, 2018). Poly (lactic-co-glycolic acid) (PLGA) nanoparticles have been approved by the US Food and Drug Administration (FDA) and the European Medicines Agency because of their excellent biocompatibility and high loading capacity for various insoluble therapies. The effectiveness of PLGA nanoparticles as nanocarriers has previously been demonstrated in many studies (Hsu et al., 2015). Therefore, PLGA nanoparticles were selected as carriers and they were loaded with PFD to evaluate the effect of inhibiting urethral stenosis through the local slow and controlled release of PFD.

In this study, PFD was selected as an antifibrosis drug, and it was combined with a PLGA nanoparticle polymer and a catheter to construct a catheter loaded with NP/PFD complexes. This catheter was then inserted into urethral stenoses in a rabbit model. The prevention and inhibition of urethral strictures were studied using histopathological and immunohistochemical analyses.

## Materials and methods

### Preparation of NP/PFD complexes

In reference to previous studies, nanoparticles were obtained by a two-emulsion method, as described in detail below. Two hundred microlitres of phosphate-buffered saline (PBS), PFD emulsified in 4 mL of dichloromethane, and 40 mg of PLGA (Mw = 40,000–75,000; Sigma-Aldrich, Saint Louis, MO, United States) were shaken in a Sonoplus HD 2070 ultrasonic homogeniser (Bandelin, Berlin, Germany) in an ice bath for 1 min. Subsequently, 9 mL of 1.5% poly (vinyl alcohol) (PVA; Mw = 14,160) was added to the mixture to form a double emulsion. The double emulsion was re-emulsified in an ultrasonic homogeniser in an ice bath for 3 min and stirred at room temperature for 24 h to completely evaporate the dichloromethane. Finally, the nanoparticles were centrifuged at 12,000 rpm at less than 4°C for 5 min, washed three times in deionised water, and suspended in deionised water (Jiang et al., 2022).

### Preparation and characterisation of catheters loaded with NP/PFD complexes

First, catheters (Fr6, 360 mm, Jiangyang Special Rubber & Plastic Products Co., LTD., Yangzhou, China) were incubated in PBS (5 mM, PH 8.5) containing dopamine hydrochloride (0.5 mg/mL). Polydopamine-modified catheters were formed by stirring at room temperature for 3 h. The PDA-modified catheters were then washed twice with deionised water and immersed in the PFD-containing nanoparticle suspension prepared in the previous step to form a catheter loaded with NP/PFD complexes. Finally, a scanning electron microscope (SEM; S-3400N; Hitachi Tokyo, Japan) was used to observe the surface morphology of the catheter loaded with the NP/PFD complexes (Jiang et al., 2022).

### Preparation of rhodamine-B-labelled NP/PFD complexes

To evaluate the distribution of nanoparticles after catheter loading, NP/PFD complexes were inserted into the urethra. Rhodamine B (Sigma-Aldrich) was used to label the NP/PFD complexes. The detailed steps are described below. Two hundred microlitres of PBS, rhodamine B, PFD emulsified in 2 mL of dichloromethane, and 20 mg of PLGA were shaken in a Sonoplus HD 2070 ultrasonic homogeniser in an ice bath for 0.5 min. Then, 4.5 mL of 1.5% PVA was added to the mixture to form a double emulsion. The double emulsion was re-emulsified in an ultrasonic homogeniser in an ice bath for 3 min and stirred at room temperature for 24 h to completely evaporate the dichloromethane. Finally, the nanoparticles were centrifuged at 12,000 rpm at less than 4°C for 5 min, washed three times in deionised water, and suspended in deionised water (Jiang et al., 2022).

### Preparation of a catheter loaded with rhodamine-B-labelled NP/PFD complexes

First, the catheters were incubated in PBS containing dopamine hydrochloride. Polydopamine-modified catheters were formed by stirring at room temperature for 3 h. The polydopamine-modified catheters were then washed twice with deionised water and immersed in the rhodamine-B-labelled NP/PFD complex suspension prepared in the previous step to form a catheter loaded with rhodamine-B-labelled NP/PFD complexes (Jiang et al., 2022).

### Detection of NP/PFD complex release *in vitro*

The amount of PFD released from the catheter loaded with the NP/PFD complexes was measured in PBS (pH 7.4). First, the 30 mm catheter was incubated in 2 mL of PBS with 0.02% w/v sodium azide and then placed in a 37°C incubator. The PBS was replaced on day 1, 3, 7, 14, 21, and 28, and the removed release solution were kept at −80°C. Finally, PFD release was measured using an enzyme-linked immunosorbent assay (BioOcean, Shoreview, MN, United States). This experiment was performed thrice (Jiang et al., 2022). In order to detect the drug loading capacity of NP/PFD, we calculated the unloaded PFD content in the supernatant of the prepared NP/PFD and the drug loading capacity of NP/PFD. The calculation formula is shown as follows.

$$\text{Drug loading }\left(\text{DL}\right)\left(\%\right)=\frac{\text{mass}\;\text{of}\;\text{PFD}-\text{mass}\;\text{of}\;\text{unloaded}\;\text{PFD}}{\text{mass}\;\text{of}\;\text{PFD}-\text{mass}\;\text{of}\;\text{unloaded}\;\text{PFD}+\text{mass}\;\text{of}\;\text{PLGA}}\times 100$$

### Detection of the cytotoxicity of catheter loaded with NP/PFD complexes

The cytotoxicity of the catheters loaded with NP/PFD complexes was determined using the Cell Counting Kit 8 (CCK8) assay. First, a 20 mm sterile catheter was cut into a 2 mm catheter and placed in a 96-well plate. Cell suspensions (SV-HUC-1, PC101) were then added to the wells (0.5 × 10^4^ cells/well), and after incubation for 24 and 48 h, 10 µL of CCK8 dye solution (10 mg/mL, Sigma-Aldrich) was added to the medium in each well. Cultivation was performed in an incubator at 37°C with 5% CO_2_ for 4 h. The absorbance of each well was measured at 450 nm using a microplate reader. Using untreated cells as a reference, the survival rate of treated cells was 100%. This experiment was performed thrice (Jiang et al., 2022).

### Establishment of animal model of urethral stricture

Twelve 20-week-old male New Zealand rabbits were used to construct the experimental animal models. The rabbits were divided into the following four groups: Sham (*n* = 3), urethral stricture (US) (*n* = 3), US + unmodified catheter (*n* = 3), and US + NP/PFD catheter (*n* = 3). All rabbits were intramuscularly injected with Sumianxin II (2 mg/kg) and Zoletil 50 (15 mg/kg). Approximately 5 min after successful anaesthesia, the rabbits were placed in the supine position on the operating table, their limbs were fixed, their perineal body hair was shaved with a special shaver, and the perineal body hair was routinely disinfected with povidone iodine. A catheter (Fr6) was inserted to drain the urine, and the assistant pulled the foreskin with a 6–0 silk thread to the cephalic side to expose the surgical site. The skin and tissue of the penis perineum were dissociated along the ventral side, the foreskin of the penis was incised longitudinally, and the subcutaneous tissue was cut sharply with microscissors under a microscope (10× light lens) to avoid blood vessels and maintain a clear surgical field of vision. The assistant pulled the skin on both sides to expose the urethra and determined that the electrocoagulation site was 2 cm from the urethral opening. An electric coagulation guidewire (LK-3, China) with set parameters was used for electrocoagulation. After the completion of electrocoagulation (10 W, 2 s), the skin and subcutaneous tissue were sutured with a 6–0 absorbable thread. All four procedures were performed by the same two urological surgeons in our research group, and the animals were intramuscularly injected with 0.5 mg of cefazoloxime for 3 consecutive days after surgery. After the operation, the rabbits were fed alone, could eat and drink normally, and moved freely. The animal study was approved by the Laboratory Animal Care and Committee of Nantong University (approval number: S20210301-991). The welfare of the experimental animals followed the Chinese National Guidelines (GB/T 35892-2018).

### Retrograde urethrography

On the 30th day after surgery, the rabbits were successfully anaesthetised using the anaesthesia method described above. They were then placed supine or inclined on the X-ray examination bed of a digital gastrointestinal contrast machine, and the irradiation window was adjusted and fixed to the lower abdomen. The Fr6 catheter was inserted approximately 1.0 cm into the outer orifice of the urethra, and ioversol and sterilised water were prepared in a 1:1 ratio as contrast agents. An empty 50 mL needle was used to push the contrast agent slowly through the catheter into the urethra, and an image was captured after complete development of the urethra and bladder. The length and inner diameter of the stenosis segment were measured.

### Histopathological and immunohistochemical staining analyses

On the 30th postoperative day, the rabbits were euthanised using excessive anaesthesia at the end of retrograde urethrography procedure. The foreskin of the penis was quickly opened by tissue clipping, and the surrounding urethral tissue was peeled off. The damaged urethral tissue was located and resected. Paraformaldehyde-fixed urethral tissues, paraffin embedding, and sectioning were performed. Tissue morphology was observed by haematoxylin and eosin (HE) staining, and the protein expression of TGF-β1 was detected by immunohistochemical staining.

### Statistical analysis

Descriptive data are presented as mean ± SD. The cytotoxicity assay analysis was performed using an unpaired Student’s *t*-test to examine the differences between groups. Statistical significance was set at *p* < 0.05.

## Results

### Characterisation and detection of catheters loaded with NP/PFD complexes

**FIGURE 1:**
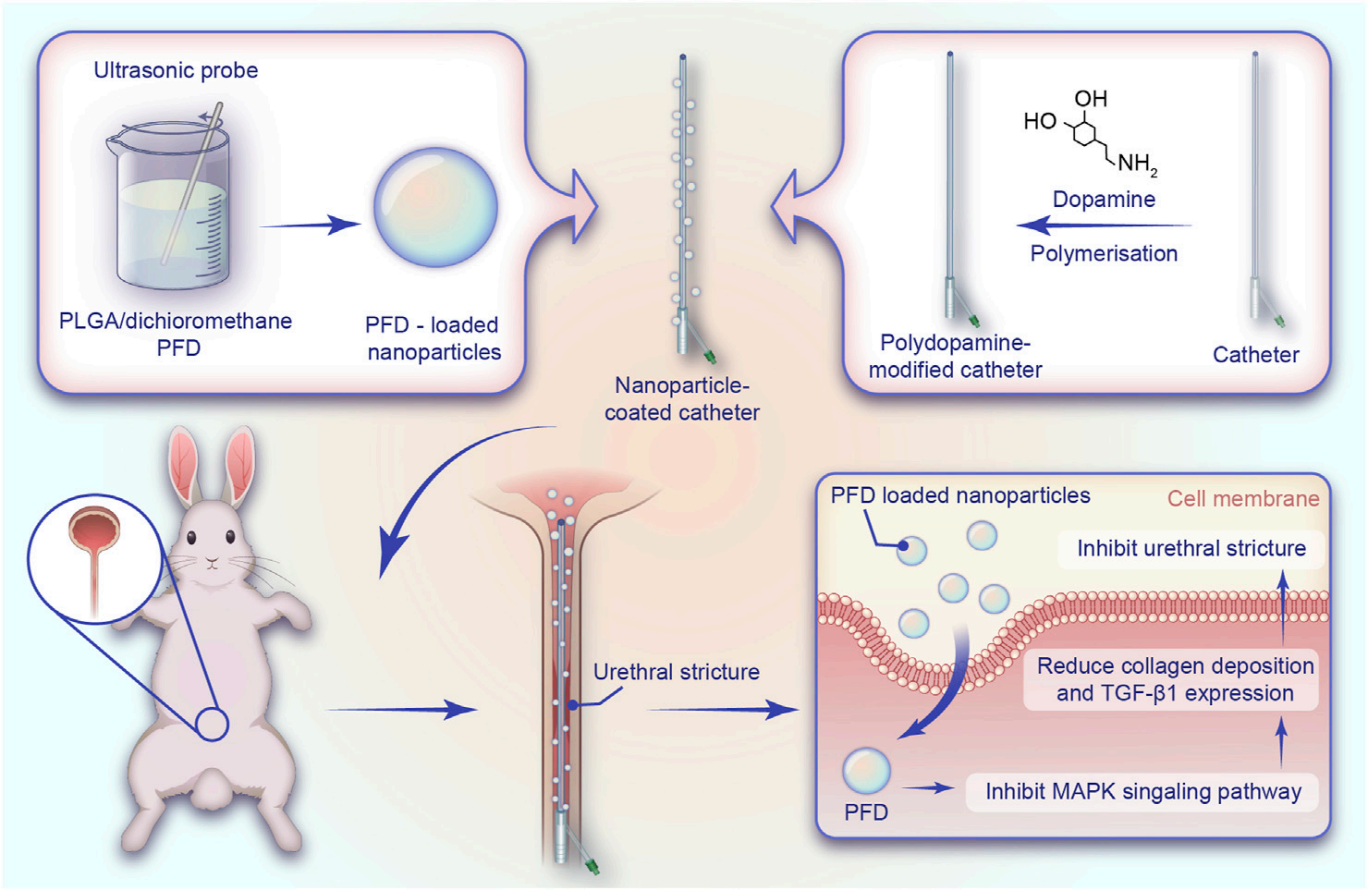
Schematic diagram of the preparation of the catheter loaded with nanoparticle/pirfenidone complexes, the experimental procedure of rabbits, and the mechanism of inhibiting urethral strictures. NP, nanoparticle; PFD, pirfenidone; PLGA, poly (lactic-co-glycolic acid).

**FIGURE 2:**
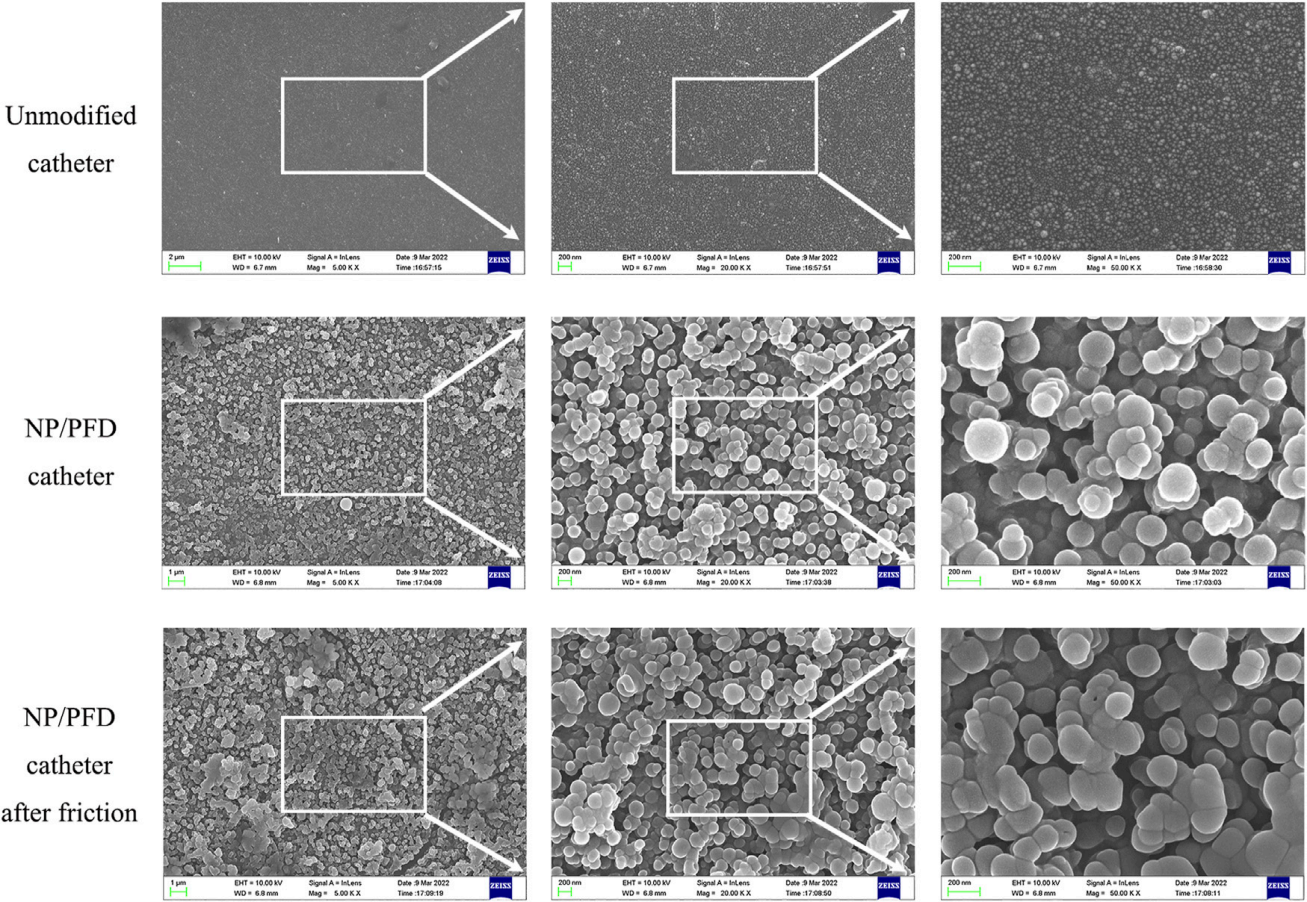
Typical scanning electron microscope images of three different forms of catheters. NP, nanoparticle; PFD, pirfenidone.

The entire preparation process of the catheter loaded with NP/PFD complexes, the general process of the experiment, and the relevant mechanism of inhibiting urethral stricture are presented as a flowchart (Figure 1). Scanning electron microscopy was used to observe the distribution of the NP/PFD complexes loaded on the catheter surface. In addition, we examined NP/PFD complex retention on the catheter surface after implantation of a catheter loaded with NP/PFD complexes into the urethra. Representative SEM images of the catheters in three different states (unmodified catheters, catheters loaded with NP/PFD complexes, and catheters loaded with NP/PFD complexes after friction) are shown in Figure 2. We found that large amounts of NP/PFD complexes remained on the catheter surface, even after catheter placement in the urethra.

### Distribution of NP/PFD complexes in the urethra

**FIGURE 3:**
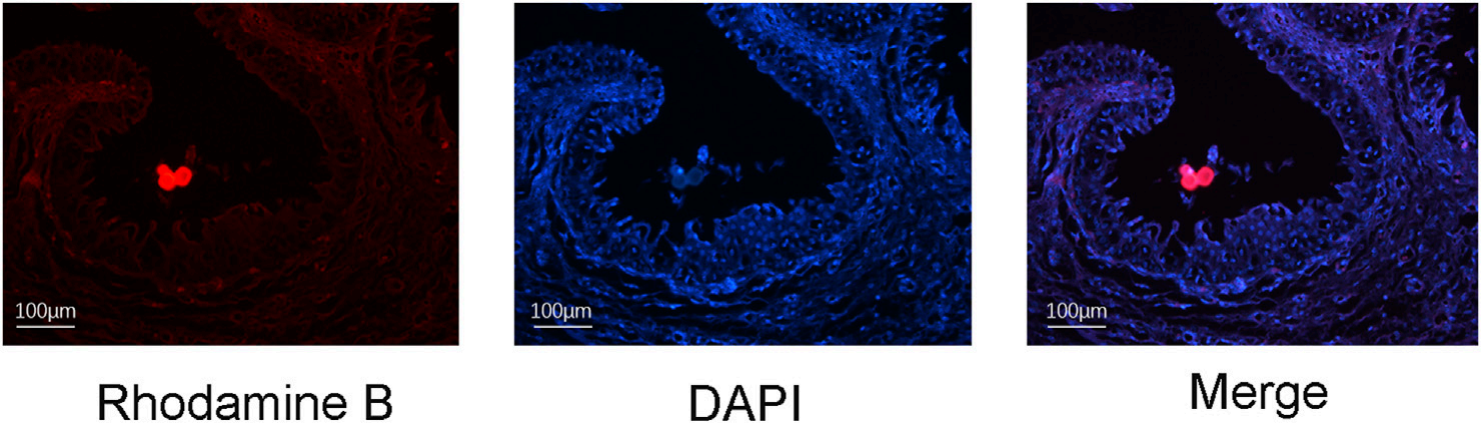
Distribution of rhodamine-B-labelled nanoparticle/pirfenidone complexes in urethral tissue 1 week after urethral placement. DAPI, 4′,6-diamidino-2-phenylindole.

One week after the placement of a rhodamine-B-labelled catheter loaded with NP/PFD complexes, we observed a large amount of red fluorescence around the urethral epithelium (Figure 3), indicating that the NP/PFD complexes on the catheter were released and diffused into the tissues around the urethra.

### Release of NP/PFD complexes *in vitro*

**FIGURE 4:**
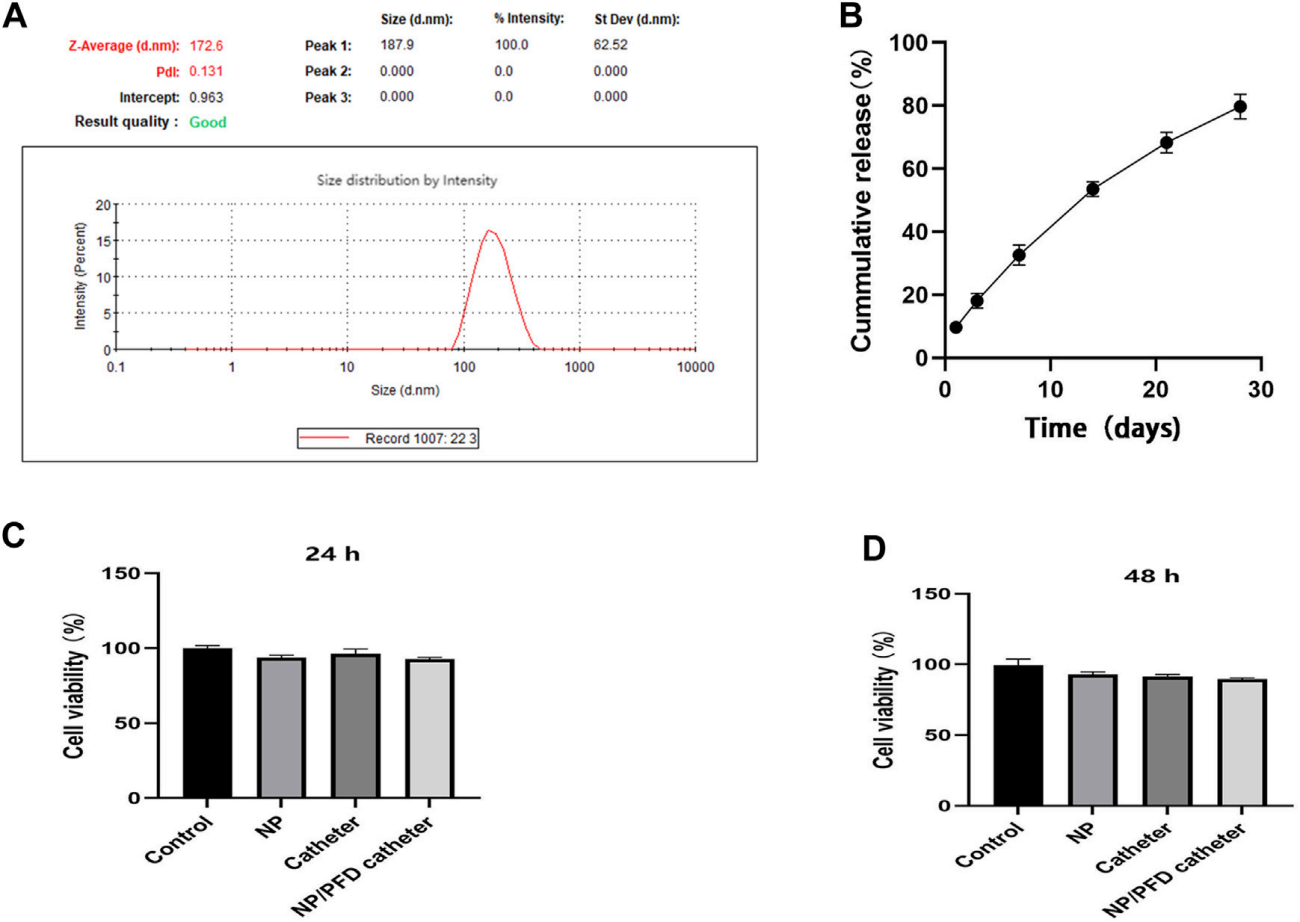
Characteristics of catheters loaded with nanoparticle/pirfenidone complexes. **(A)** Size of nanoparticle/pirfenidone (NP/PFD) complexes. **(B)** *In vitro* release profile of a catheter loaded with NP/PFD complexes. **(C,D)** Cytotoxic effects of different catheters and nanoparticles on urethral epithelial cells after co-culture for 24 and 48 h.

We selected biodegradable PLGA to synthesise nanoparticles to sustainably release PFD and inhibit urethral strictures. The diameter of these NP/PFD complexes was determined by dynamic light scattering (DLS), and they were found to have an average diameter of 187.90 ± 62.52 nm (As shown in Figure 4A). In addition, we determined the *in vitro* release profiles of the catheters loaded with NP/PFD complexes. Within the first 3 days, we found that approximately 20% of the PFD was released from the catheters loaded with NP/PFD complexes. Finally, approximately 80% of PFD was slowly and continuously released from the catheter loaded with NP/PFD complexes within 30 days (As shown in Figure 4B). These results suggest that the slow release of PFD can be achieved using catheters loaded with NP/PFD complexes. In addition, the DL% is 14.12% ± 0.81%.

### Cytotoxicity of catheters loaded with NP/PFD complexes

To evaluate the cytotoxicity of catheters loaded with NP/PFD complexes, a CCK8 assay was performed using cultured human ureteral epithelial cells. Compared with untreated cultured cells, the unmodified catheters, NPs, and catheters loaded with NP/PFD complexes showed no significant cytotoxicity after 24 and 48 h of co-culture (As shown in Figures 4C, D).

### Rabbit model of urethral injury

**FIGURE 5:**
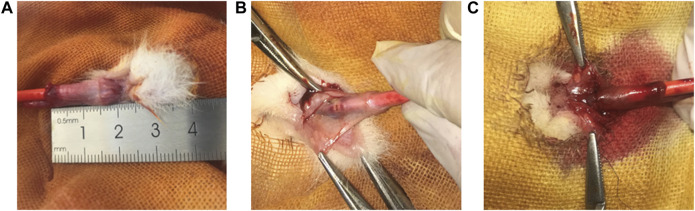
Rabbit modelling experiment operation image. **(A)** Image of normal urethra in rabbits. **(B)** Image of electrical coagulation injury of rabbit urethra. **(C)** Image of a sutured rabbit urethra in a model of electrocoagulation injury.

Rabbit models of normal urethra, urethral electrical injury, normal catheter after urethral electrical injury, and catheter containing NP/PFD complexes after urethral electrical injury were established respectively (Figure 5).

### Retrograde urethrography in rabbits

**FIGURE 6:**
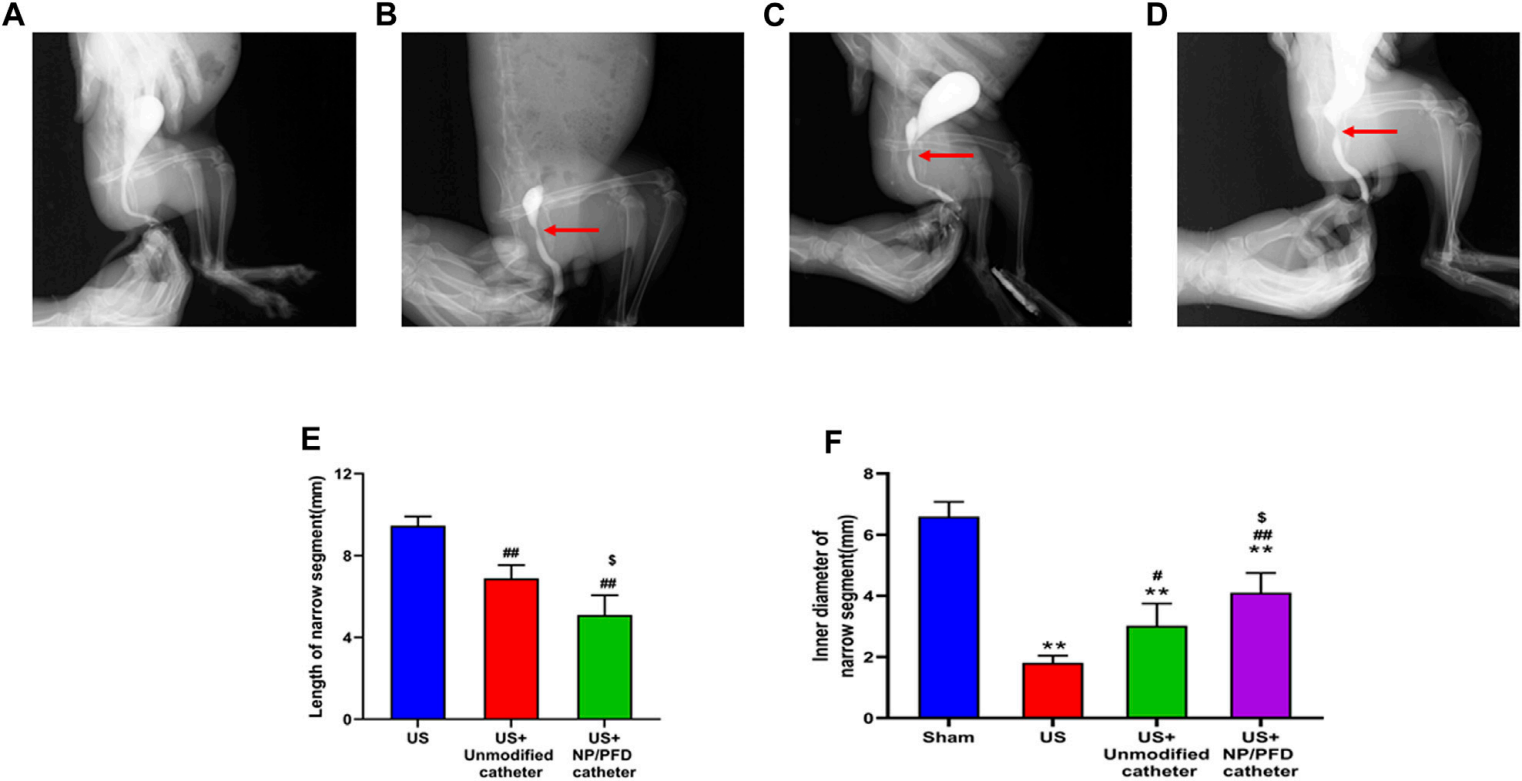
Retrograde urethrography in a rabbit model. **(A)** Urethrogram images of rabbits in the sham group. **(B)** Urethrography images of rabbits in the US group. **(C)** Urethrogram images of rabbits in the US + unmodified catheter group. **(D)** Urethrogram images of rabbits with US + NP/PFD complexes loaded catheter group. US, urethral stricture; NP/PFD, nanoparticle/pirfenidone. **(E)** Length of narrow segments between different groups. **(F)** The inner diameter of the stenosis segment between the different groups. (Compared with Sham group, *p < 0.05, **p < 0.01; Compared with the US group, #*p* < 0.05, ##*p* < 0.01; Compared with the US + Unmodified catheter group, $*p* < 0.05.)

Retrograde urethrography showed that the length of the urethral stricture narrow segment in the US group was 9.46 ± 0.45 mm, US + Unmodified catheter group was 6.88 ± 0.66 mm, US + NP/PFD catheter group was 5.10 ± 0.96 mm. Besides, the inner diameter of urethral stricture was 6.59 ± 0.48 mm in sham group, 1.81 ± 0.23 mm in US group, 3.02 ± 0.73 mm in US + Unmodified catheter group, and 4.10 ± 0.65 mm in US + NP/PFD catheter group. According to the results, it can be concluded that the catheter loaded with NP/PFD complexes can reduce the length of the urethral stricture segment and improve the inner diameter of the urethral stricture segment (As shown in Figure 6).

### Haematoxylin and eosin and immunohistochemical staining analyses

**FIGURE 7:**
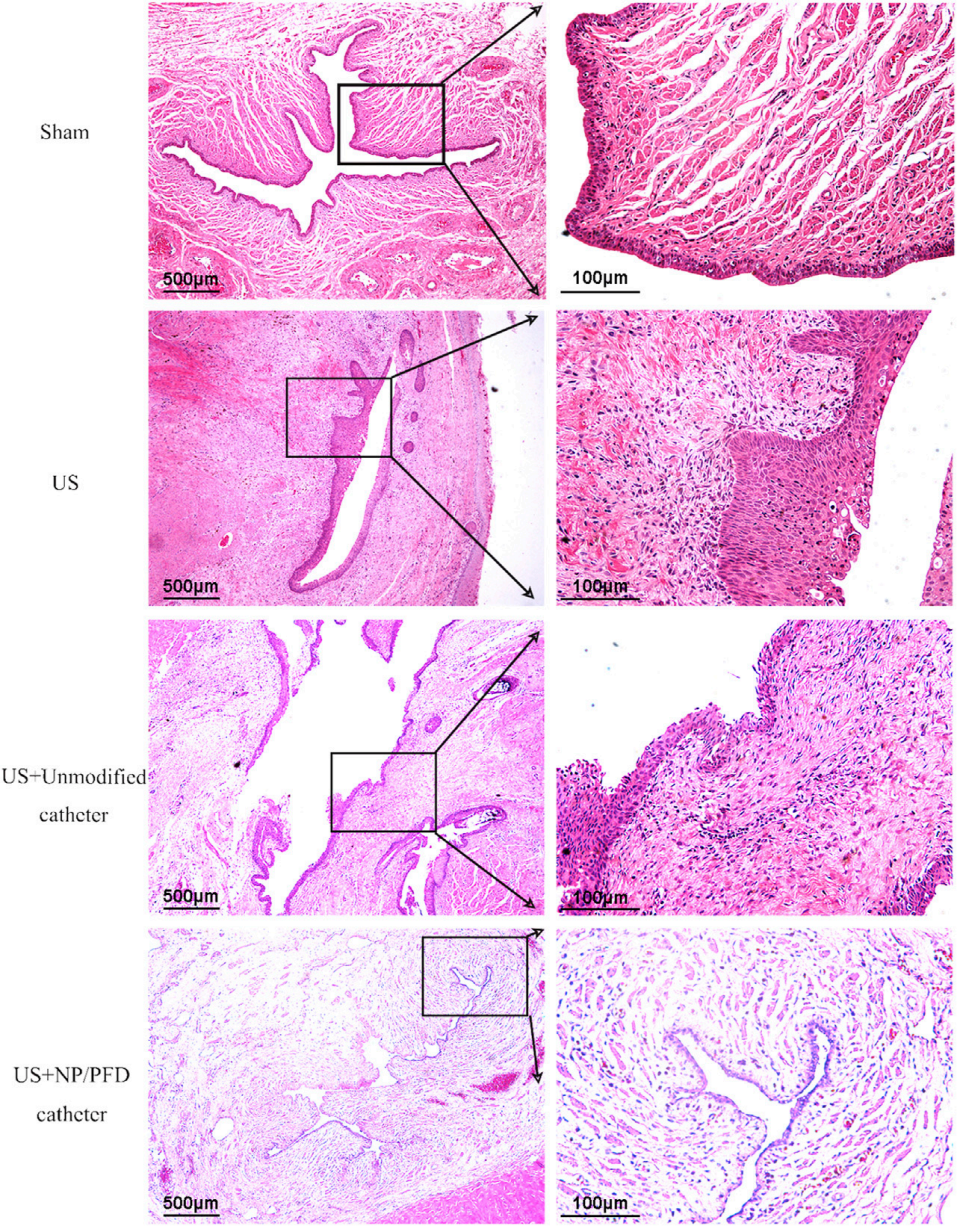
Photomicrograph of haematoxylin staining. The urethral lumen of the US group was significantly narrowed, and the diameter of the urethral lumen of the US + NP/PFD group was larger than that of the US group and the US + unmodified catheter group. US, urethral stricture; NP/PFD, nanoparticle/pirfenidone.

**FIGURE 8:**
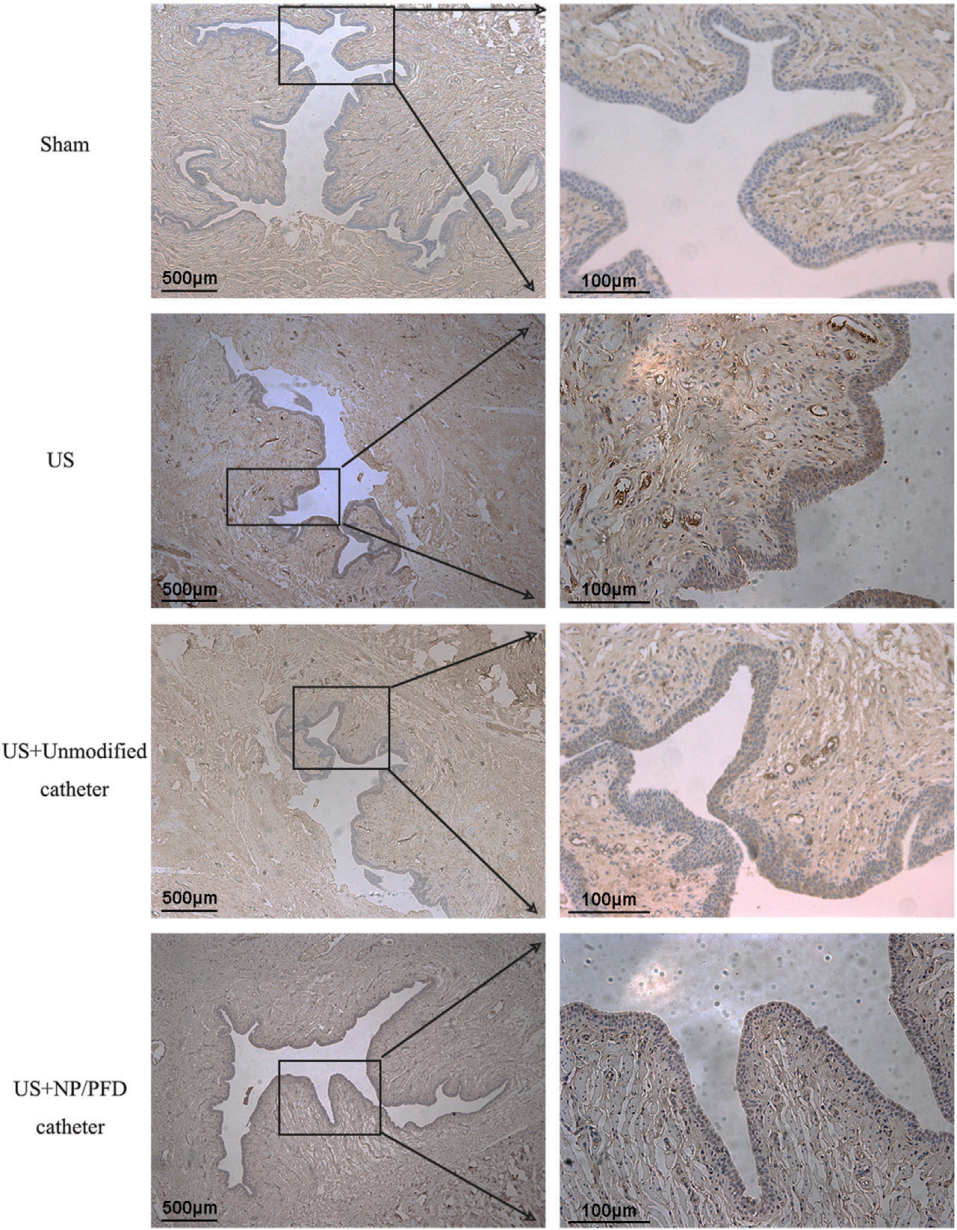
Photomicrograph of immunohistochemical staining. The expression level of TGF-β1 was lower in the US + NP/PFD catheter group than in the US group and the US + unmodified catheter group. US, urethral stricture; NP/PFD, nanoparticle/pirfenidone.

HE staining of the transverse sections of urethras in the US group showed severe urethral stricture. Urethral epithelial cell proliferation was evident, the lumen was significantly narrowed, the number of inflammatory cells increased in the surrounding tissue, and the cell arrangement was disordered. Simultaneously, urethral stricture was improved by placing an unmodified catheter, whereas urethral stricture was significantly improved in rabbits after placing the catheter loaded with NP/PFD complexes. The lumen of the urethras in the US + NP/PFD group showed mild epithelial cell hyperplasia, the peritubular tissue showed little inflammatory cell infiltration, and the cell arrangement was regular (Figure 7). Immunohistochemistry experiments showed differences in the expression levels of TGF-β1 between the different groups. The expression level of TGF-β1 was lower in the periurethral tissues of catheters loaded with NP/PFD complexes than in those of the US group. Therefore, PFD may inhibit tissue fibrosis by inhibiting the expression of TGF-β1 (Figure 8).

## Discussion

Urethral strictures easily recur and often form intractable scars requiring repeated urethral dilation or surgical treatment. This has become a serious problem in urology. In recent years, research on the prevention of urethral strictures has increased. For example, rapamycin (Chong et al., 2011) prevents the formation of urethral strictures in rabbits by inhibiting fibroblast proliferation and collagen expression, which may be related to the TGF-β/Smad signalling pathway (Li et al., 2022). Docetaxel (Fu et al., 2014) inhibits urethral strictures by inhibiting fibrosis and collagen coagulation. Mitomycin C (Chang et al., 2015) may be considered as an adjunct treatment option for urethral strictures after urethral injury. These studies provide a basis for the development of clinical drugs for the prevention and treatment of urethral strictures. In this study, the antifibrosis drug, PFD, was selected as the research object to explore its influence on the formation of urethral strictures after urethral injury in rabbits. The results of retrograde urethrography, histopathology, and immunohistochemistry showed that PFD can effectively inhibit fibrosis after urethral injury, and therefore, it may be a new therapeutic choice to prevent urethral strictures.

PFD is an antifibrosis drug that has been approved by the FDA for the treatment of idiopathic pulmonary fibrosis owing to its effective inhibition of liver and lung fibrosis (Hall et al., 2018). The formation of a urethral scar and the recurrence of strictures are related to the fibrosis of the injured tissue during healing. Previous studies have shown that the MAPK signalling pathway plays an important role in the transformation of TGF-β1-induced epithelial cells into myofibroblasts, and PFD can produce antifibrotic effects by antagonising the MAPK signalling pathway (Jiang et al., 2022). In this study, reverse urethrography showed that PFD enlarged the lumen of injured urethras; HE staining showed that PFD relieved urethral tissue fibrosis and collagen deposition; and immunohistochemical analysis showed that PFD inhibited the expression of TGF-β1 in injured tissue. These results provide a basis for the study of PFD in the prevention of urethral stenosis after injury and the associated mechanism of action.

PFD is prone to degradation after direct exposure to the tissue environment, and the loss of its sustained-release properties prevents the sustained release of the drug and limits its therapeutic effectiveness. Therefore, a delivery system that delivers the drug slowly and consistently is required. PLGA copolymers exhibit excellent biocompatibility and biodegradability (Guo et al., 2023), and unique physical and chemical properties, making them among the most popular and effective polymers for drug delivery (Hashemi et al., 2020). In this study, a PLGA nanoparticle drug delivery system was used as the carrier. A catheter loaded with NP/PFD complexes was formed by wrapping it with PFD. The morphological characteristics of the catheter loaded with NP/PFD complexes, the amount of PFD released into the epithelium, and the cytotoxicity of the catheter were verified by scanning electron microscopy, fluorescence detection of the catheter loaded with rhodamine-B-labelled NP/PFD complexes, detection of released PFD, and cytotoxicity detection using the CCK8 assay. The results showed that the constructed catheter loaded with NP/PFD complexes has many advantages, such as non-toxicity and controllable drug release. It is worth emphasizing that in this study, in order to ensure the residual amount of NP/PFD complexes on the surface of the catheter, we modified the catheter with polydopamine before loading the NP/PFD complexes. Previous studies have shown that polydopamine has strong adhesion ability and a variety of biochemical properties (Jin et al., 2020). Therefore, more NP/PFD complexes can be aggregated and delivered to the urethral tissue, so that it can be taken up by various cells such as urethral epithelial cells and fibroblasts.

To date, various urethral injury stenosis models have been established; however, there is no unified method. Previous studies on urethral stricture modelling have included the establishment of a canine urethral stricture model with an F10 paediatric electric incision mirror and a 2 mm ring electrode. Scar tissue formation was observed at the urethral injury sites in all animals 1 month after surgery (Li et al., 2022). In another study, a surgical electrotome and guidewire were used to coagulate the electrode and burn the urethra to establish an injury-stenosis model (Shinchi et al., 2019). In addition, some studies have simulated urethral stenosis after urethral injury using a simple incision and suturing of the urethra (Chen et al., 2018). Urethrography and urethroscopy are often used to evaluate the inner diameter of the urethra to determine the success of establishing a urethral stricture model. Simultaneously, histopathological examination and immunohistochemical staining can be used to observe changes in the urethral lumen, urethral epithelial hyperplasia, and the expression of genes related to fibrosis. Guided by the theoretical basis of the abovementioned studies, we adopted the electric coagulation and burning method to establish a rabbit urethral injury stenosis model that can be reduced, simple, and easy to establish. Urethrography and histopathology confirmed that this method resulted in post-injury stenosis and could be widely applied to urethral stenosis modelling.

Previous reports have not clarified the specific molecular mechanism whereby PFD inhibits fibrosis, but most studies have concluded that TGF-β1 may be the key molecule in the action of PFD (Meng et al., 2016; Shinde et al., 2017). According to relevant reports, PFD reduces the infiltration of M2 macrophages and inhibits the activation of TGF-β1/Smad3 signalling to improve radiation-induced lung fibrosis (Ying et al., 2021). In this study, we found that PFD inhibits TGF-β1 expression, which may be one of the potential mechanisms whereby it inhibits the fibrotic process in the formation of urethral strictures. There are some limitations in this study. Owing to the insufficient number of experimental animals, the specific mechanism whereby urethral strictures were prevented with a catheter loaded with NP/PFD complexes was not elucidated, and subsequent experiments need to be performed to further explore this mechanism.

## Conclusion

A catheter loaded with NP/PFD complexes can prevent and reduce urethral strictures, which provides more references for the development of clinical drugs for the treatment of urethral strictures. The rabbit urethral stricture modelling method we used is simple to operate. The stricture model was established successfully and can be used as an effective method for animal urethral stricture modelling.

## Data Availability

The original contributions presented in the study are included in the article/Supplementary material, further inquiries can be directed to the corresponding authors.

## Abbreviations

NP/PFD, nanoparticle/pirfenidone; US, urethral stricture; TGF-β1, transforming growth factor β1; ECM, extracellular matrix; PLGA, poly (lactic-co-glycolic acid); PBS, phosphate-buffered saline; rpm, revolutions per minute; SEM, scanning electron microscope; PVA, poly (vinyl alcohol); CCK8, Cell Counting Kit 8.
